# Age and sex, not APOE, drive brain volume changes in humanized APOE mice: A model of aging and risk, not disease

**DOI:** 10.1002/alz70856_107547

**Published:** 2026-01-09

**Authors:** Adam C. Raikes, Avnish Bhattrai, Tian Wang, Roberta Diaz Brinton

**Affiliations:** ^1^ University of Arizona, Tucson, AZ, USA

## Abstract

**Background:**

Whole brain and hippocampal atrophy are key structural features of late‐onset Alzheimer's disease (LOAD) and common clinical trial endpoints. The APOE ε4 allele is the strongest genetic risk factor, with ε4/ε4 genotype linked to the greatest atrophy rates. Structural differences in preclinical APOE models remain underreported, limiting assessments of translatability. Here, we considered a humanized APOE (hAPOE) mouse model of AD, and assessed age, sex, and genotype effects on volumetric brain measurements in a voxel‐wise manner.

**Method:**

High‐resolution ex‐vivo T2w‐RARE MRIs were acquired from male and female hAPOEε3/ε3, ε3/ε4, and ε4/ε4 mice aged 6, 9, 15, and 24 months. Brains were warped to a study‐specific template, and voxel‐wise analyses were performed on relative Jacobians, reflecting local differences independent of total brain size. The model included mean‐centered age, sex, and APOE genotype (ε4/ε4 vs. ε3/ε4; ε3/ε3 vs. ε4+). Significant voxels were thresholded at FDR‐corrected *p* < 0.001 and classified using the Dorr atlas.

**Result:**

Significant clusters of voxels were identified for age and sex but not APOE genotype. Age‐related volume decreases were observed in cortical regions, cerebellar cortex, striatum, and thalamus, while increases occurred in deep midbrain structures, including the medulla and pons, and white matter tracts such as the corpus callosum. Males had greater volume in midbrain structures, cerebellar cortex, striata, and subcortical gray matter, while females had greater cortical volume, consistent with other rodent models.

**Conclusion:**

Our findings leverage a large dataset (*n* = 159) of high‐resolution MRIs in mice spanning 6‐24 months (human age range: ∼34‐70). Age‐related changes indicate cortical contraction and midbrain/white matter expansion. Observed sex differences align with prior mouse studies: females exhibit greater cortical volume, while males show greater subcortical volume. Notably, there were identifiable areas of the hippocampi where volume was smaller in females than males, consistent with human studies. No genotypic effects survived multiple comparison correction, suggesting that age and sex drive volumetric changes rather than APOE and that the ε4 allele in this model does not result in the phenotypic atrophy observed in LOAD. This model reflects aging and neurodegenerative risk rather than disease, providing insights into brain structural dynamics over the lifespan.

